# A Cross-Sectional Analytic Study of Postpartum Health Care Service Utilization in the Philippines

**DOI:** 10.1371/journal.pone.0085627

**Published:** 2014-01-20

**Authors:** Tadashi Yamashita, Sherri Ann Suplido, Cecilia Ladines-Llave, Yuko Tanaka, Naomi Senba, Hiroya Matsuo

**Affiliations:** 1 Kobe City College of Nursing, Nursing, Kobe, Japan; 2 Department of International Health, Kobe University Graduate School of Health Sciences, Kobe, Japan; 3 Department of Obstetrics and Gynecology of the Philippine General Hospital, Manila, Philippines; 4 University of the Philippines, College of Medicine, Manila, Philippines; 5 Department of Nursing, Seisen University, Hikone, Japan; 6 Department of Maternity Nursing, Kobe University Graduate School of Health Sciences, Kobe, Japan; Kenya Medical Research Institute - Wellcome Trust Research Programme, Kenya

## Abstract

**Background:**

The maternal mortality ratio in the Philippines remains high; thus, it will be difficult to achieve the Millennium Development Goals 5 by 2015. Approximately two-thirds of all maternal deaths occur during the postpartum period. Therefore, we conducted the present study to examine the current state of postpartum health care service utilization in the Philippines, and identify challenges to accessing postpartum care.

**Methods:**

A questionnaire and knowledge test were distributed to postpartum women in the Philippines. The questionnaire collected demographical characteristics and information about their utilization of health care services during pregnancy and the postpartum period. The knowledge test consisted of 11 questions regarding 6 topics related to possible physical and mental symptoms after delivery. Sixty-four questionnaires and knowledge tests were analyzed.

**Results:**

The mean time of first postpartum health care visit was 5.1±5.2 days after delivery. Postpartum utilization of health care services was significantly correlated with delivery location (*P*<0.01). Women who delivered at home had a lower rate of postpartum health care service utilization than women who delivered at medical facilities. The majority of participants scored low on the knowledge test.

**Conclusion:**

We found inadequate postpartum health care service utilization, especially for women who delivered at home. Our results also suggest that postpartum women lack knowledge about postpartum health concerns. In the Philippines, Barangay health workers may play a role in educating postpartum women regarding health care service utilization to improve their knowledge of possible concerns and their overall utilization of health care services.

## Introduction

Through the Millennium Development Goals 5 (MDGs5), countries have committed to reducing the maternal mortality ratio (MMR) by three-quarters between 1990 and 2015 [1]. In the Philippines, the MMR has dropped from 170 (per 100,000 live births) in 1990 to 99 in 2010 (120 in 2000), but the rate of annual change is slowing down [2]–[4]. If the MMR remains high in the Philippines, it will be difficult to accomplish the MDGs5 by 2015. Four measures have been undertaken to improve the MMR in the Philippines: 1) the implementation of a capacity-enhancement project for midwives in maternal and newborn care [5], 2) education by hospitals on the Safe Motherhood Policy [6], 3) the establishment of facilities to provide emergency obstetric care for every 125,000 members of the population [7], and 4) the recommendation of at least four visits for antenatal care [8]. These measures have contributed to the improvement of the MMR in the Philippines. However, the major causes of maternal death in the Philippines in 2009 were complications occurring during the course of pregnancy, delivery, and the postpartum period (41.0%) such as pregnancy-induced hypertension (PIH; 32.1%) and postpartum hemorrhage (PPH; 17.9%) [9]–[11]. This indicates that many maternal deaths occur not only during pregnancy and delivery but also during the postpartum period [12] [13]. In fact, approximately two-thirds of all maternal deaths occur during the postpartum period [14] [15]. Therefore, providing postpartum health care services may be important for reducing the MMR in the Philippines. However, there are no reports regarding utilization of postpartum health care services or the challenges associated with seeking such services in the Philippines.

Thus, we conducted the present study to examine the current status of postpartum health care service utilization in the Philippines and elucidate the factors that affect the utilization of postpartum health care services.

## Methods

### Participants

Seventy-seven postpartum women from 3 hours to six weeks post-delivery with no complications were enrolled in this study, which was conducted from January to March, 2013. The questionnaire and knowledge tests were distributed to women who received postpartum checkups at Philippine General Hospital (PGH) and those who participated in a postpartum health education seminar at Muntinlupa City Hall. Participants were recruited using a two-stage stratified random sampling method. The seminar addressed several issues, including current status and problems related to maternal death in postpartum women. Thirteen incomplete questionnaires and knowledge tests were excluded; thus, 64 questionnaires and knowledge tests (PGH: 22, Muntinlupa: 42) were included in the analyses. In this study, health care services were defined as medical care such as prescriptions, blood pressure measurement, and urinalysis, as well as preventive care (e.g., checkups and breastfeeding education).

### Self-report Questionnaire

The questionnaire comprised 3 sections. The first section assessed characteristics including age, delivery location, past delivery history, number of household members, health insurance status, and annual family income. The second section assessed the utilization of health care services during pregnancy (HS-pre): number of HS-pre, the time of the first HS-pre visit, place HS-pre was received, and difficulties with receiving HS-pre. The third section assessed the utilization of postpartum health care services (HS-post): number of HS-post, the time of first HS-post visit, place HS-post was received, difficulties with receiving HS-post, family support, possession of the Maternal and Child Health Handbook (MCH), and the place where the MCH had been obtained.

### Postpartum Health Concerns Test

We developed a test to evaluate postpartum women’s knowledge of postpartum health; its reliability was examined by Philippine and Japanese researchers in obstetrics/gynecology and midwifery. The test consisted of 11 questions on six areas of possible postpartum physical and mental health concerns: 1) genital bleeding, 2) gastrointestinal symptoms, 3) blood pressure, 4) cranial nerve symptoms, 5) infection, and 6) mental state. Participants were asked to respond to each symptom by choosing one of two responses: “seek immediate medical attention” or “do not seek immediate medical attention.” We determined the accuracy rate and calculated the knowledge level of the postpartum women based on correct and incorrect responses.

The questionnaire and knowledge test were translated from English into Tagalog by a Filipino translator with knowledge of obstetrics and gynecology.

### Statistics

To analyze questionnaire data, the sample was divided into two groups: those who had received one or more HS-post and those who had received no HS-post. A chi-square test was used to compare participant characteristics (age, delivery location, delivery history, number of household members, health insurance status, and annual family income), HS-pre utilization (number of HS-pre visits, time of first HS-pre visit, place HS-pre was received, and difficulties with receiving HS-pre), and knowledge test accuracy rate between these two groups. Data for age, number of family members, annual family income, and number of HS-pre were divided into above average and below average for analysis. Analyses were two-tailed, with a *P* value of <0.01 considered significant. Data were analyzed using SPSS Statistics 18 (IBM, Inc.).

All participants provided written informed consent for all procedures associated with the study. This research was approved by the Ethics Committee of the Kobe University Graduate School of Health Sciences Ethics Board and the Human Research Ethics Committee of the Philippine General Hospital.

## Results

### Participant Characteristics

Participant characteristics are summarized in Table 1. The mean postpartum period was 13.6±9.3 days (mean ± SD), and ranged from 0 to 38. Mean age of participants was 26.0±6.9 years, ranging from 16 to 45. Delivery locations were divided into four categories: health center (31.3%), hospital or clinic (50.0%), home (15.6%), and other (3.1%). Annual family income (Philippine pesos; php) was classified into four categories: under 100,000 (45.3%), 100,001–250,000 (18.8%), 250,001–500,000 (3.1%), and over 500,001 (1.6%). The majority (77.2%) of participants had no health insurance, and most (98.4%) reported having family support for childrearing.

**Table 1:** Participant characteristics, Philippines.

	Total N (%)	Mean ± SD	Range
**Postpartum period (days)**	62	13.6±9.3	0–38
**Age (years)**	61	26.0±6.9	16–45
<20	11 (18.0)		
20–34	41 (67.2)		
>35	9 (14.8)		
**Number of family members**	60	5.6±2.2	2–10
**Past delivery history**	64	2.3±1.9	1–11
**Delivery location**	64		
Health Center	20 (31.3)		
Hospital or clinic	32 (50.0)		
Home	10 (15.6)		
Other	2 (3.1)		
**Annual family income (php** a **)**	44		
Under 100,000	29 (45.3)		
100,001–250,000	12 (18.8)		
250,001–500,000	2 (3.1)		
Over 500,001	1 (1.6)		
**Health insurance**	57		
Have	11 (19.3)		
Do not have	44 (77.2)		
Do not know	2 (3.5)		
**Family childrearing support**	63		
Yes	62 (98.4)		
No	1 (1.6)		

^a^ php: Philippine Pesos.

### Utilization of Health Care Services during Pregnancy (HS-pre)

The mean number of HS-pre received was 4.9±3.8, ranging from 0 to 13. The majority of women had their first HS-pre visit within 11 weeks of pregnancy (33.9%), followed by 20–27 (26.8%), 12–19 (14.3%), ≥28 (16.1%), and unknown (8.9%) The majority of women (83.9%) reported no difficulty receiving HS-pre (Table 2).

**Table 2:** Utilization of health care services during pregnancy, Philippines.

	TotalN (%)	Mean ± SD	Range
**Number of HS-pre** a	64	4.9±3.8	0–13
**Time of first HS-pre visit**	56		
Within 11 weeks	19 (33.9)		
12–19 weeks	8 (14.3)		
20–27 weeks	15 (26.8)		
28 or more weeks	9 (16.1)		
Do not know	5 (8.9)		
**HS-pre provider**	62		
Obstetrician or healthcenter doctor	36 (58.1)		
Nurse	6 (9.7)		
Midwife	19 (30.6)		
Other	1 (1.6)		
**Place HS-pre was received**	61		
Hospital or clinic	21 (34.4)		
Health center	38 (62.3)		
Home	2 (3.3)		
**Difficulty with receiving HS-pre**	62		
Yes	10 (16.1)		
No	52 (83.9)		
**Reasons for difficulty**	21		
CostAccess	10 (47.7)5 (23.8)		
Lack of family support	2 (9.5)		
Poor physical condition	0 (0)		
Other	4 (19.0)		

^a^ HS-pre: Health care services during pregnancy.

### Postpartum Utilization of Health Care Services (HS-post)

The mean time of first postpartum health care visit was 5.1±5.2 days after delivery. Time of the first HS-post visit was divided into 3 categories: within 3–23 hours (13.2%), 24–48 hours (39.4%), and 3–41 days (47.4%). The majority of women (82.6%) reported no difficulty receiving HS-post.

More than half of the participants (60.7%) had not received the Maternal and Child Health handbook (MCH). Of those who had, 26.9% received it from a hospital or clinic, 53.8% from a health center, and 19.2% from another location. Women reported using the MCH at their check-ups (47.8%), their baby’s check-ups (8.7%), their own or their baby’s check-ups (39.1%), or at another time (4.3%) (Table 3).

**Table 3:** Utilization of postpartum health care services, Philippines.

	TotalN (%)	Mean ± SD	Range	
**Number of HS-post** a	64	1.8±2.8	0–13	
**Time of first HS-post visit** **(days)**	38	5.1±5.2	0–18	
4–23 hours	5 (13.2)			
24–48 hours	15 (39.4)			
3–41 days	18 (47.4)			
**HS-post provider**	44			
Obstetrician or healthcenter doctor	27 (61.4)			
Nurse	6 (13.6)			
Midwife	8 (18.2)			
Other	3 (6.8)			
**Place HS-post was received**	38			
Hospital or clinic	25 (52.1)			
Health center	15 (31.3)			
Home	3 (6.3)			
Other	5 (10.4)			
**Difficulty receiving HS-post**	46			
Yes	8 (17.4)			
No	38 (82.6)			
**Reasons for difficulty**	18			
Cost	12 (66.7)			
Access	3 (16.7)			
Lack of family support	1 (5.6)			
Poor women’s physical condition	1 (5.6)			
Other	1 (5.6)			
**Possession of MCH** b	61			
Have	24 (39.3)			
Do not have	37 (60.7)			
**Place MCH was obtained**	26			
Hospital or clinic	7 (26.9)			
Health center	14 (53.8)			
Other	5 (19.2)			
**Use of MCH**	23			
Checkups for mothers	11 (47.8)			
Checkups for children	2 (8.7)			
Checkups for mothers andchildren	9 (39.1)			
Other	1 (4.3)			

^a^ HS-post: postpartum health care services.

^b^ MCH: Maternal and Child Health Handbook.

### Factors Affecting HS-post Utilization

HS-post utilization was significantly correlated with delivery location (*P*<0.01). Women who delivered at home had a lower rate of HS-post utilization than those who delivered at medical facilities (i.e., hospital, clinic, or health center). There was no significant difference between the utilization of HS-post and participant characteristics or the utilization of HS-pre. One trend did suggest that health insurance, participant age, family size, and time of first HS-pre visit may be related to HS-post utilization (Table 4).

**Table 4:** Factors affecting postpartum health care service utilization in the Philippines.

	Total N	Utilization (+)^c^	Utilization (−)^d^	*P*-value
		n (%)	n (%)	
**Delivery location**				
Health Center	20	13 (65.0)	7 (35.0)	.007*
Hospital or clinic	32	24 (75.0)	8 (25.0)	
Home	10	2 (20.0)	8 (80.0)	
**Health insurance**				0.335
Have	11	8 (72.7)	3 (27.3)	
Do not have	44	25 (56.8)	19 (43.2)	
**Age (years)**				
<20	11	9 (81.8)	2 (18.2)	0.219
21–34	41	22 (53.7)	19 (46.3)	
>35	9	6 (66.7)	3 (33.3)	
**Number of family members**				
<4	22	15 (68.2)	7 (31.8)	0.325
>5	38	21 (55.3)	17 (44.7)	
**Annual family income (php)**				
<100,000	29	19 (65.5)	10 (34.5)	0.431
>100,001	15	8 (53.3)	7 (46.7)	
**Past delivery history**				
Primiparous	28	18 (64.3)	10 (35.7)	0.728
Multiparous	35	21 (60.0)	14 (40.0)	
**Number of HS-pre** a				
<4	17	11 (64.7)	6 (35.3)	0.822
>5	39	24 (61.5)	15 (38.5)	
**Time of first HS-pre visit (weeks of pregnancy)**				
<27	27	15 (55.6)	12 (44.4)	0.147
>28	24	18 (75.0)	6 (25.0)	
**Difficulty receiving HS-pre**				
Yes	10	7 (70.0)	3 (30.0)	0.612
No	52	32 (61.5)	20 (38.5)	
**Possession of MCH** b				
Have	22	15 (68.2)	7 (31.8)	0.795
Do not have	37	24 (64.9)	13 (35.1)	
**Place MCH was obtained**				
Hospital or clinic	6	5 (83.3)	1 (16.7)	0.67
Health center	12	8 (66.7)	4 (33.3)	
Other	5	3 (60.0)	2 (40.0)	
**Family childrearing support**				
Yes	56	34 (60.7)	22 (39.3)	0.424
No	1	1 (100.0)	0 (0.0)	

Significant (*P*<0.01).

^a^ HS-pre: Health care services during pregnancy,

^b^ MCH: Maternal and Child Health Handbook.

^c^ Utilization (+): Postpartum women who utilized one or more postpartum health care services.

^d^ Utilization (−): Postpartum women who had no utilization of postpartum health care services.

### Knowledge Regarding Postpartum Health Concerns

**Figure 1:**
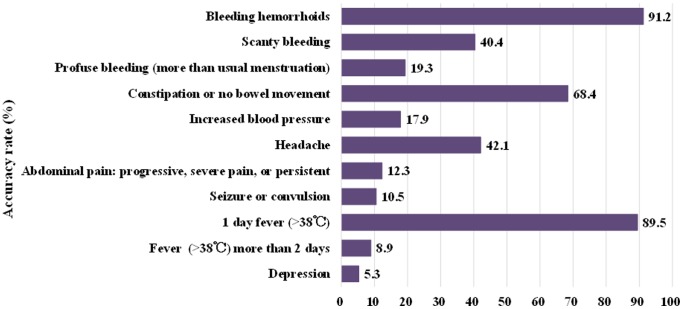
Accuracy rate of knowledge test in postpartum women, Philippines. This figure shows accuracy rate of knowledge test on postpartum health issues.

The majority of participants scored low on the knowledge test. The accuracy rate on the knowledge test ranged from 5.3% (regarding depression) to 91.2% (regarding bleeding hemorrhoids). Specific accuracy rates for each question are depicted in Figure 1. There was no significant difference between HS-post utilization and accuracy rate on the knowledge test.

## Discussion

As the first survey of postpartum health care service utilization and its limitations conducted in the Philippines, this study found the following: participants did not receive postpartum health care services at the appropriate time, those who delivered at home utilized fewer health care services after delivery compared to those who delivered at medical facilities, and they had a poor overall understanding of postpartum health issues.

In the Philippines, postpartum health care guidance may be inadequate due to the large number of deliveries, few skilled birth attendants, and short post-delivery stays (i.e., only 24 hours) at perinatal medical facilities. As a result, postpartum women may have less knowledge of postpartum issues and receive fewer postpartum health care services [16]. WHO reported that the first 24 to 48 hours are the most critical time for postpartum woman [17]. It indicates that postpartum women should receive first HS-post within 2 days after delivery. Actually, this survey revealed that 47.4% of postpartum women attended their first HS-post in 3–41 days after delivery, which is very late. This length of time before the first HS-post visit and poor overall understanding of postpartum health issues might contribute to late detection of postpartum health problems. Interestingly, most women in this study indicated that they had no difficulty utilizing HS-post. It may be possible that most postpartum women in the Philippines have access to HS-post; however, they may not utilize them at the appropriate time due to a lack of understanding about postpartum health issues. Therefore, it is important to address women’s knowledge of the postpartum period and related health issues and provide adequate postpartum health care guidance.

Our study supports previous research that has found that HS-post utilization might be related to postpartum women’s characteristics (e.g., age, family income, delivery history, and health insurance status, among others) and HS-pre utilization [18]–[29]. Therefore, it is important that health care providers such as midwives, nurses, obstetricians, and gynecologists provide HS-post based on women’s characteristics and HS-pre utilization history. In the Philippines, 40% of women deliver at home with the assistance of traditional birth attendants rather than medical professionals due to a lack of money and access to medical facilities [30]–[32]. In the present study, women who delivered at home had lower rates of HS-post utilization than women who delivered at medical facilities. Postpartum women who deliver at home may not have the opportunity to utilize health care services. Further research will be necessary to focus on postpartum health care services for women who deliver at home. In the Philippines, there are few skilled birth attendants and a general lack of guidance regarding health care after delivery due to the large number of deliveries and short post-delivery stays at perinatal medical facilities. Therefore, it may be effective to utilize Barangay health workers (BHWs; community volunteers who provide services such as maternal, newborn, and child health care in Filipino neighborhoods) as health care providers [33]–[35].

Our study had several limitations. Our sample size was small, the sample region was limited, and some participants reported lower-than-average income levels. However, this cross-sectional study was the first analysis of the current status of postpartum health care service utilization and its limitations conducted in the Philippines.

In conclusion, we have demonstrated inadequate utilization of postpartum health care services, both in terms of access to care (especially for women who delivered at home) and utilization of postpartum services at the appropriate time. We also demonstrated that postpartum women lacked knowledge about potential postpartum health issues. In the Philippines, BHWs could play an important role in educating postpartum women on the utilization of postpartum health care services based on women’s knowledge level, individual characteristics, and health care service utilization history.
